# New model for predicting preterm delivery during the second trimester of pregnancy

**DOI:** 10.1038/s41598-017-11286-x

**Published:** 2017-09-12

**Authors:** Ya-zhi Zhu, Guo-qin Peng, Gui-xiang Tian, Xue-ling Qu, Shui-yuan Xiao

**Affiliations:** 1 Department of Social Medicine and Health Management, Xiangya School of Public Health, Central South University, Changsha, 410078 China; 2 Department of Medical Affairs, The Second Xiangya Hospital, Central South University, Changsha, Hunan 410011 China; 3 Department of Obstetrics and Gynecology, Xiangya Hospital, Central South University, Changsha, Hunan 410008 China; 4 Department of ultrasound, The Second Xiangya Hospital, Central South University, Changsha, Hunan 410011 China; 5 Department of ultrasound, Weihai municipal hospital, Weihai, Shandong 264200 China

## Abstract

In this study, a new model for predicting preterm delivery (PD) was proposed. The primary model was constructed using ten selected variables, as previously defined in seventeen different studies. The ability of the model to predict PD was evaluated using the combined measurement from these variables. Therefore, a prospective investigation was performed by enrolling 130 pregnant patients whose gestational ages varied from 17^+0^ to 28^+6^ weeks. The patients underwent epidemiological surveys and ultrasonographic measurements of their cervixes, and cervicovaginal fluid and serum were collected during a routine speculum examination performed by the managing gynecologist. The results showed eight significant variables were included in the present analysis, and combination of the positive variables indicated an increased probability of PD in pregnant patients. The accuracy for predicting PD were as follows: one positive – 42.9%; two positives – 75.0%; three positives – 81.8% and four positives – 100.0%. In particular, the combination of ≥2× positives had the best predictive value, with a relatively high sensitivity (82.6%), specificity (88.1%) and accuracy rate (79.2%), and was considered the cut-off point for predicting PD. In conclusion, the new model provides a useful reference for evaluating the risk of PD in clinical cases.

## Introduction

Preterm delivery (PD) remains a global problem associated with perinatal morbidity, including low birth weight, growth retardation and irreversible damage to the nervous system^1^. The incidence of PD ranges from 5% to 15% worldwide, indicating that approximately 15 million preterm babies were born before 37 completed weeks (W) of gestation, which is the second leading cause of perinatal death^2^. In America, 6.14 infant deaths per 1,000 live births and 35.2% of infant deaths were related to PD in 2010^3^. In China, there was a 7.1% incidence of preterm births, and 7769 preterm births occurred between 28 and 37 W of pregnancy in 2011^4^. In addition, one survey showed an increased mortality associated with PD with an average annual growth rate of 1.52% since 1996, accounting for 22.6% of infant mortality in 2013. Therefore PD is considered the leading cause of infant death^5^.

Approximately thirty years ago, a risk scoring system was proposed for predicting preterm birth, providing a significant reference for further study^6^. To date, many studies have been performed regarding the prediction of preterm birth. Although these studies have resulted in improved prediction of PD and a decrease in the number of premature births, at present, the accuracy of predicting preterm births is still a puzzle because of many factors that contribute to the outcome of PD. These factors include a previous history of PD, gestational age, pregnancy complications, psychological and genetic factors^7^, maternal obesity^8, 9^, placenta previa^10^, fat-to-placenta strain ratio value^11^, serum relaxin^12^, insulin-like growth factor-binding protein-1^13^, interleukin-1β (IL-1β)^14^, thioredoxin and interleukin 1 receptor antagonist^15^, and fetal fibronectin levels and cervical length measurements^16, 17^. However, some of these factors have shortcomings with respect to sensitivity or specificity, which affects the accuracy of PD prediction. Based on previous studies, and seeking to improve the sensitivity and specificity for predicting PD, the present study proposes a new prediction model for premature birth.

## Results

### Selection of test variables

**Figure 1:**
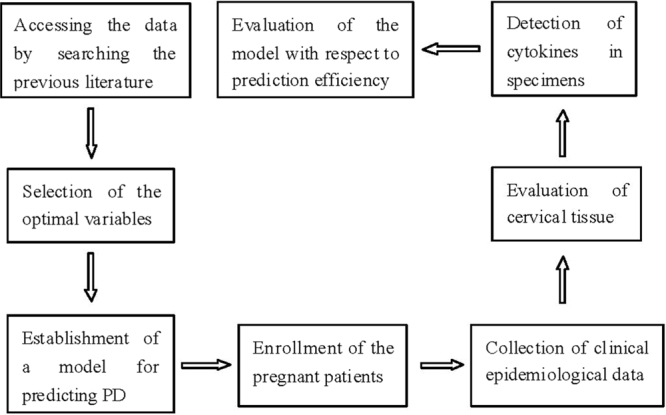
Strategy of constructing and verifying the model.

According to the study strategy described in the materials and methods section (Fig. 1), ten variables were selected from the previous seventeen single-center or multicenter studies; these studies provided a large enough sample size for evaluating cut-off values, sensitivity and specificity. These variables were classified as epidemiological indices, cervical characteristics and cytokine level in cervicovaginal fluid or serum. Positive or negative results were judged by the mean cut-off values, indicating an increased risk of PD. In addition, the mean cut-off values, sensitivity and specificity were calculated by evaluating the difference from the original literature. The results of the test variables used in the model are shown in detail in Table 1.

**Table 1:** Variables for predicting preterm delivery in the second trimester of pregnancy.

Test variables (units)	Value of cut-off^M^	Sensitivity^T^ (%)	Specificity^T^ (%)	PD %^T^ (n)	Type of literature
History of preterm delivery^14, 18, 19^	Yes	51.6	78.1	15.7 (896)	3× Single center
Prepregnancy BMI^18, 20^ (kg/m^2^)	<20	35.6	70.0	17.5 (3333)	1× Single center 1× Multicenter^S^
Use of tocolytic agents^21^	Yes	55.4	79.5	15.4 (719)	1× Multicenter
Cervix tissue elasticity^22, 23^ (Blue area-%)	<11.0	79.2	85.3	15.1 (397)	2× Single center
Densitometry of cervix^24–26^ (Mean gray value)	≤7.7	84.0	75.0	37.5 (231)	3× Single center
Cervical dilatation^21^ (cm)	1~3	63.8	67.6	15.4 (719)	1× Multicenter
CL-single gestation^18, 27, 28^ (mm)	≤30.9	69.8	87.2	14.3 (906)	3× Single center
CL-twin gestations^29^ (mm)	≤25.0	64.0	93.0	9.6 (3213)	1× Multicenter^S^
Fetal fibronectin in CVF^21, 25^ (ng/mL)	≥50	59.1	83.0	17.6 (880)	1× Multicenter 1× Single center
Serum MIF^30, 31^ (ng/mL)	>9.2	53.3	75.4	32.9 (365)	2× Single center
IL-1β in CVF^14, 32, 33^ (pg/mL)	>55	77.0	53.0	27.9 (559)	3× Single center

Notes: n, the number of sample; PD%, the percentage of occurring PD; M, the data were shown as the mean level if there was a difference existing in several previous studies; T, the total levels of several single or multicenter studies were evaluated by the corresponding definitions of sensitivity, specificity and PD rate; S, the secondary analysis of the Maternal-Fetal Medicine Units Network or a meta-analysis of the original article. Abbreviations: BMI, body mass index; CL, cervical length; CVF, cervicovaginal fluid; MIF, macrophage migration inhibitory factor; IL-1β, interleukin-1β.

### Characteristic of pregnant patients

**Figure 2:**
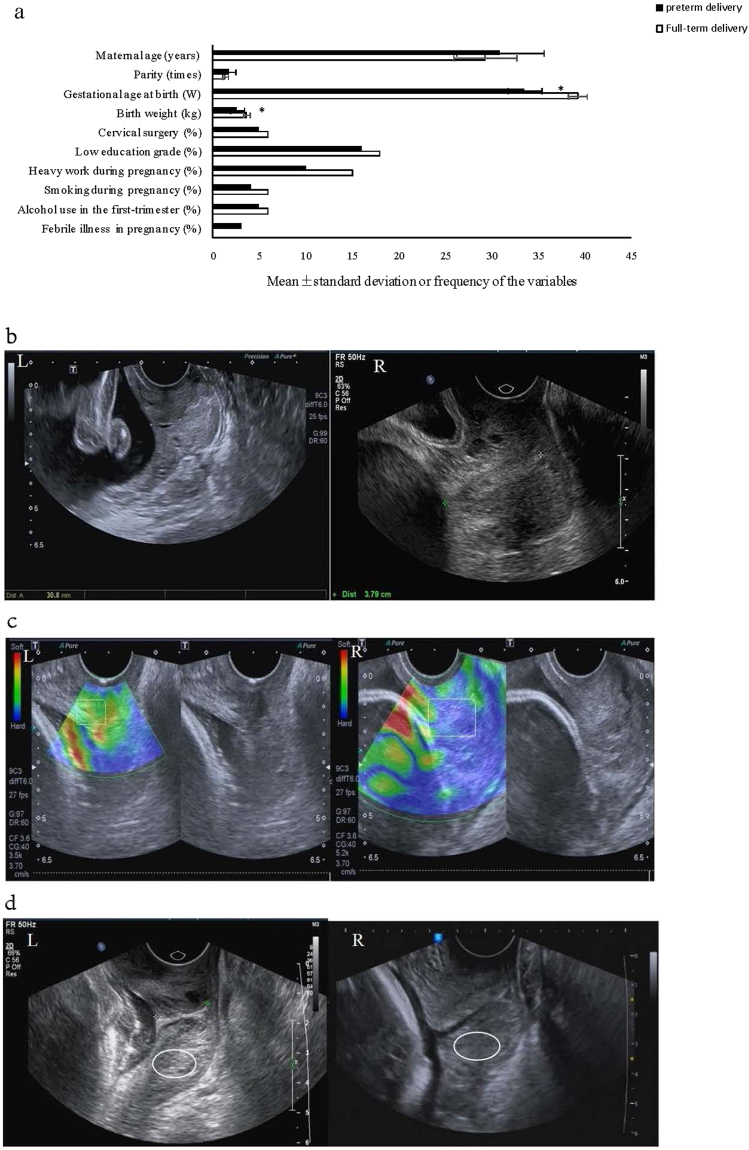
Characteristics of the pregnant patients. Note: (**a**) Basic characteristics of patients with preterm birth and full-term delivery. *P < 0.05 indicates a significant difference between preterm delivery and full-term delivery; (**b**) Cervical length as determined by transvaginal ultrasound in pregnant patients. Left (L) shows a shortened cervix (30.8 mm), Right (R) shows a normal cervical length (37.9 mm); (**c**) Blue area for cervical tissue as determined by sonoelastography in pregnant patients, the blue area indicates the stiffness of the cervical tissue. Left shows the percentage of blue area as an ROI (10.8%) that indicates a soft elasticity of the cervix, Right shows the percentage of blue area as an ROI (29.7%) that indicates relatively stiff cervical tissue; (**d**) Mean gray value of the sagittal transvaginal view in pregnant patients. Left shows a decreased echogenicity and grayscale value of 4.85, Right shows a normal echogenicity and grayscale value of 11.78.

Of 186 pregnant women enrolled in the study, 56 patients were excluded based on exclusion criteria during pregnancy. Exclusion criteria included triple gestation (n = 2), threat of abortion (n = 4), serious infection of the genital tract (n = 4), pregnancy-induced hypertension (n = 4), fetal anomaly (n = 4), fetal cytomegalovirus infection (n = 2), and 36 patients lost to follow-up due to refuse cooperation with the gynecologist’s investigation (n = 11) and uninformed absence in scheduled visiting to participating hospitals (n = 9) were also excluded from the present study. The remaining 130 patients were included in the study, and 46 (35.4%) had spontaneous preterm birth before 37 weeks of gestation. 36 patients of which delivered at 32 to 36 weeks for 78.3%, 6 patients delivered at 28 to 31 weeks for 13.0% and 4 patients delivered before 28 weeks for 8.7%. There was no significant difference between the groups in terms of maternal age, parity, the rate of singleton or twin gestations, ratio of nulliparous vs. multiparous, cervical surgery, education grade, work or lifestyle or febrile illness during pregnancy (*P* > 0.05). However, cases in the preterm group had a significantly lower gestational age (33.50 vs. 39.20 W) and lower birth weight (2,573 vs 3,628 g) than those in the full-term group. Detailed demographic and clinical information are summarized in Table 2 and Fig. 2.

**Table 2:** Basic characteristics of patients with preterm birth or full-term delivery.

Epidemiological variables (units)	Preterm delivery <37 W (n = 46)	Full-term delivery (n = 84)	Statistical method	*P* value
Maternal age (years)	30.82 ± 4.70	29.24 ± 3.35	T test	0.654
Parity (times)	1.75 ± 0.71	1.32 ± 0.27		0.594
Gestational age at birth (W)	33.50 ± 1.82	39.20 ± 1.05		0.001*
Birth weight (g)	2,573 ± 741	3,628 ± 360		0.004*
Ratio of singleton vs twin gestations	8.2: 1.0	27.0: 1.0		0.203
Ratio of nulliparous vs multiparous	0.77: 1.0	1.4: 1.0		0.105
^a^Cervical surgery (%)	5 (10.9)	6 (7.1)	χ^2^ test	0.465
^b^Low education grade (%)	16 (34.8)	18 (21.4)		0.098
Heavy work during pregnancy (%)	10 (21.7)	15 (17.9)		0.591
Smoking during pregnancy (%)	4 (8.7)	6 (7.1)		0.751
Alcohol use in the first-trimester (%)	5 (10.9)	6 (7.1)		0.465
Febrile illness in pregnancy (%)	3 (6.5)	0 (0)	Correction χ^2^ test	0.079

Notes: T test, χ^2^ test and Correction χ^2^ test were applied to compare the difference of quantitative variables, qualitative variables (theoretical frequency ≥5) and theoretical frequency (1~5), respectively; *P < 0.05 indicates a significant difference between PD and full-term delivery; ^a^Cervical surgery indicates cervical conization or loop electrosurgical excision procedure cervical surgery; b, Low education grade indicates ≤12 years compulsory education.

### Distribution of test variables

**Figure 3:**
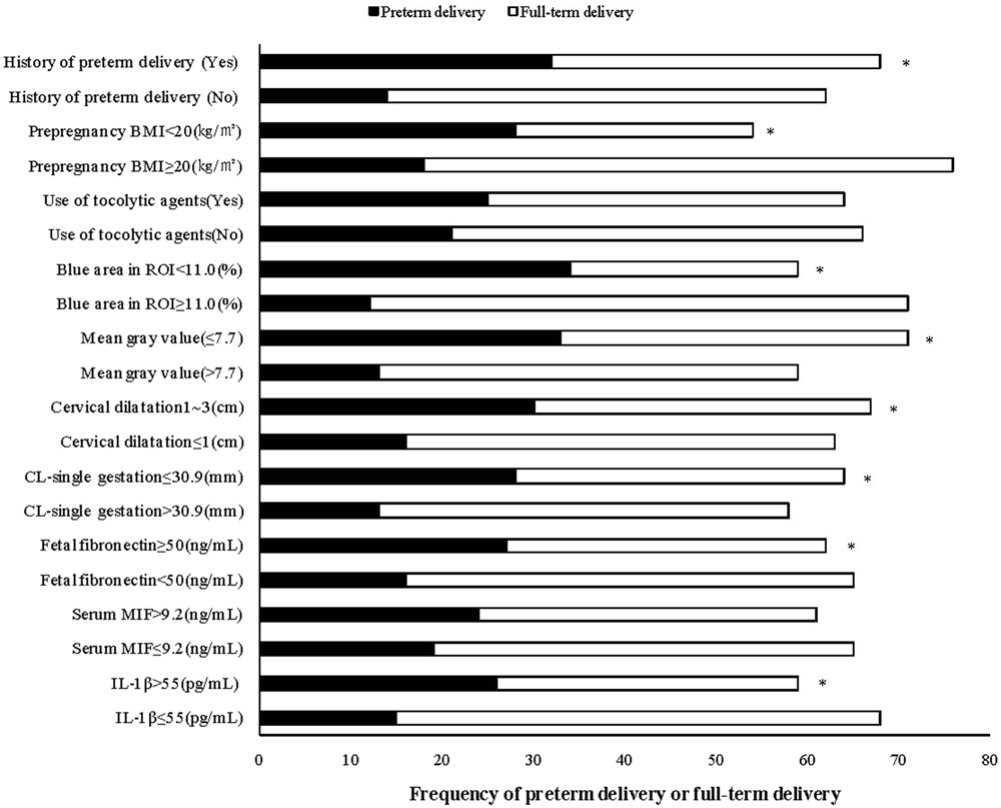
Distribution of test variables between preterm delivery and full-term delivery. Note: *P < 0.05 indicates a significant difference between preterm delivery and full-term delivery; Cervical length with twin gestations was not shown owing to the small sample size.

Statistical evaluation of the test variables was performed by comparing their positive proportions between PD and full-term delivery. A higher proportion of the following eight variables was present in the PD group than that of the full-term delivery group (*P* < 0.05): history of preterm delivery, prepregnancy BMI, the proportion of blue area, mean gray value, cervical dilatation, CL with singleton gestations, levels of fetal fibronectin and IL-1β in CVF. These were significant variables for evaluating PD in the present study. The difference between variables such as the use of tocolytic agents, CL with twin gestations and serum MIF was not significant (*P* > 0.05). Details are shown in Table 3 and Fig. 3.

**Table 3:** Distribution of test variables between preterm delivery and full-term delivery.

Test variables	Value of cut-off	n	Preterm delivery (%)	Full-term delivery (%)	*P* value
History of preterm delivery	Yes	68	32 (69.6)	36 (42.9)	0.004**
	No	62	14 (30.4)	48 (57.1)	
Prepregnancy BMI (kg/m^2^)	<20	43	28 (60.9)	26 (30.9)	0.001**
	≥20	87	18 (39.1)	58 (69.1)	
Use of tocolytic agents	Yes	61	25 (54.3)	39 (46.4)	0.388
	No	69	21 (45.7)	45 (53.6)	
Blue area in ROI (%)	<11.0	59	34 (73.9)	25 (29.8)	0.000**
	≥11.0	71	12 (26.1)	59 (70.2)	
Mean gray value (amplitude)	≤7.7	70	33 (71.7)	38 (45.2)	0.004**
	>7.7	60	13 (28.3)	46 (54.8)	
Cervical dilatation (cm)	1~3	67	30 (65.2)	37 (44.0)	0.021*
	≤1	63	16 (34.8)	47 (56.0)	
CL-single gestation (mm)	≤30.9	64	28 (68.3)	36 (44.4)	0.013*
	>30.9	58	13 (31.7)	45 (55.6)	
CL-twin gestations (mm)	≤25.0	5	3 (−)	2 (−)	1.000^#^
	>25.0	3	2 (−)	1 (−)	
^a^Fetal fibronectin (ng/mL)	≥50	62	27 (62.8)	35 (41.7)	0.024*
	<50	65	16 (37.2)	49 (58.3)	
^b^Serum MIF (ng/mL)	>9.2	61	24 (55.8)	37 (44.6)	0.231
	≤9.2	65	19 (44.2)	46 (55.4)	
^a^IL-1β (pg/mL)	>55	59	26 (63.4)	33 (38.4)	0.008**
	≤55	68	15 (36.6)	53 (61.6)	

Notes: ROI, a rectangular region of interest, indicating the midsection region in the posterior wall of the cervix; *P < 0.05 or **P < 0.01 for χ^2^ test; ^#^ *P* value for Fisher’s exact test; a, 3 CVF samples missing; b, 4 specimens of serum missing.

### Evaluation of the model

**Figure 4:**
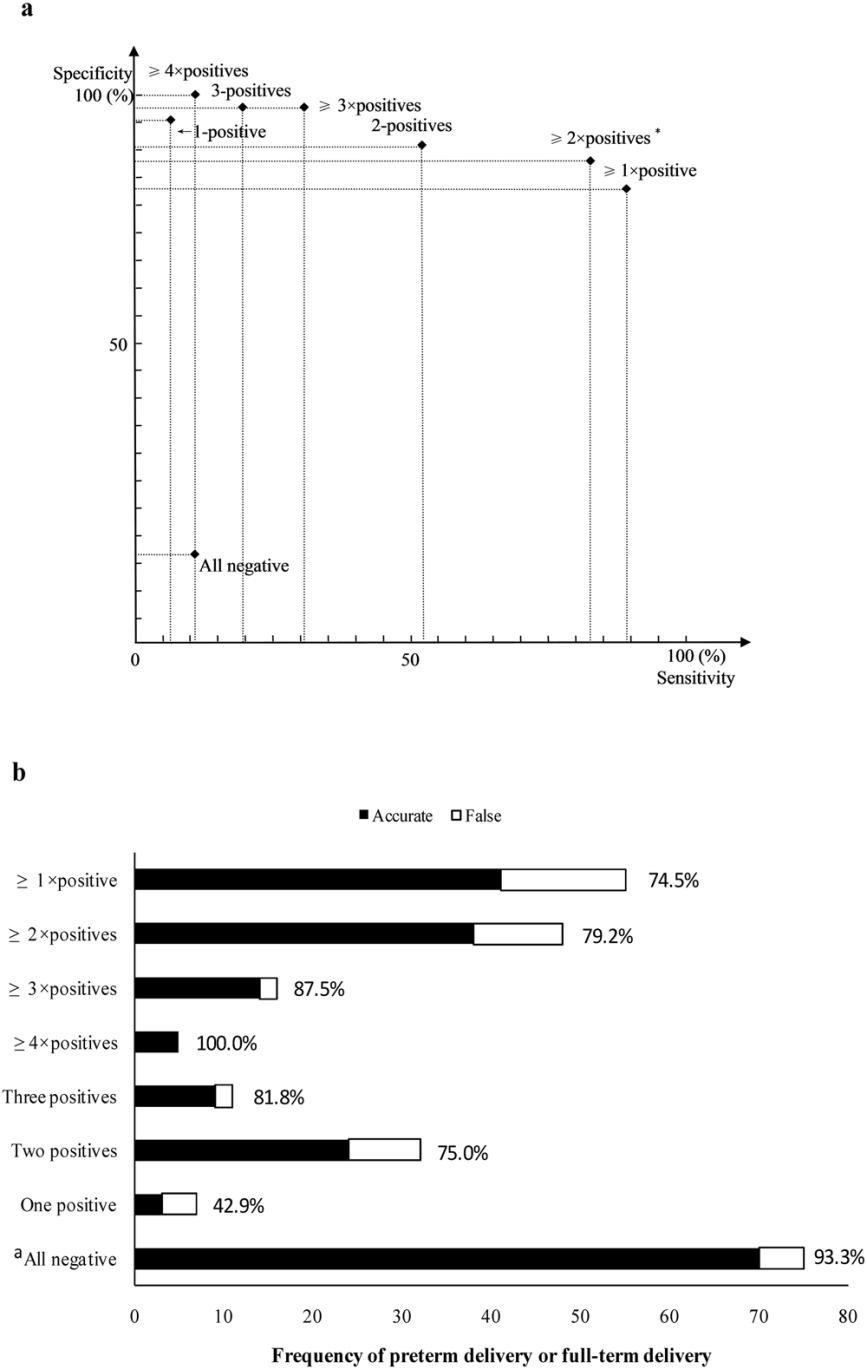
Evaluation of the model by sensitivity, specificity and accuracy. Note: (**a**) Sensitivity and specificity in single or combination of variables. The intersection of the abscissa and ordinate is represented as the characteristics of sensitivity and specificity in case of single variables or a combination of different variables. *Indicating the cutoff point of evaluating the present model; (**b**) Accuracy rate of the combination of different variables. ^a^The accuracy rate indicates the percentage of full-term delivery in pregnant patients.

Eight variables were used to evaluate the prediction efficiency of the model. The results showed a prediction efficiency of ≥1× positive, ≥2× positives, ≥3× positives, and ≥4× positives that was higher than that of all negative, one positive, two positives and three positives. Furthermore, the accuracy rate of predicting PD showed an increased trend with increasing positive variables represented as ≥1×, ≥2×, ≥3× or ≥4×. Conversely, the sensitivity showed a decreasing trend, while the specificity was still at relatively high levels. Based on the comprehensive evaluation of the accurate rate, sensitivity and specificity in different combinations of positive variables, the optimal cut-off point of the model was selected as “≥2× positives” because of the relatively high characteristics in all aspects. Details are shown in Table 4 and Fig. 4.

**Table 4:** Evaluation of the model by different combinations of test variables.

Combination of variables	Count of occurring	Preterm delivery (n)	Full-term delivery (n)	Sensitivity (%)	Specificity (%)	Accurate rate (%)
All negative	75	5	70	10.9	16.7	93.3^a^
One positive	7	3	4	6.5	95.2	42.9^b^
Two positives	32	24	8	52.2	90.5	75.0^b^
Three positives	11	9	2	19.6	97.6	81.8^b^
≥4× positives	5	5	0	10.9	100.0	100.0^b^
≥3× positives	16	14	2	30.4	97.6	87.5^b^
≥2× positives	48	38	10	82.6	88.1	79.2^b^
≥1× positive	55	41	14	89.1	83.3	74.5^b^

Notes: ^a^The accuracy rate indicates the percentage of full-term deliveries; ^b^The accuracy rate indicates the percentage of preterm deliveries.

## Discussion

A two-step strategy of establishing and verifying a predictive model for preterm delivery in the second trimester of pregnancy was proposed in this study. First, a primary model of predicting PD was proposed using a combination of test variables originating from previous studies. In the primary model, ten variables from the eighteen previous studies were selected, including three dimensions in pregnant patients represented as epidemiological indices, morphology and tissue characteristics of the cervix and inflammatory cytokines in the CVF or serum. Subsequently, the model was evaluated using a non-probability sample of 130 pregnant patients. Generally, the results of validating the model showed eight significant variables for inclusion in the present analysis. The variables of “use of tocolytic agents, serum MIF and CL in twin gestations” were excluded owing to the lack of statistical significance or small sample size. Furthermore, the combination analysis of positive variables showed that ≥2× positive variables existing in pregnant patients had a relatively high sensitivity (82.6%), specificity (88.1%) and accuracy rate (79.2%) in predicting PD, which is considered a cut-off point for predicting the occurrence of PD. Interestingly, all negative variables in the model had a high probability of association with full-term delivery, which is represented by an accuracy rate of 93.3%.

The significant association of the eight variables in the present model with PD occurrence was consistent with the conclusions from the previous studies. However, the inclusion of multiple combinations of these variables in the model showed a higher sensitivity and specificity than single or double combination of these variables in previous studies. For example, our results of 82.6% sensitivity in the combination of ≥2× positive variables was improved compared to a 33.3% sensitivity of a short cervix as a predictor of PD described by Lee *et al*.^18^ and was also higher than the 63.8% or 43.9% sensitivity predicted by cervical dilatation or fetal fibronectin alone^21^. In addition, the finding that there was no significant association with PD and the “use of tocolytic agents or serum MIF” in our model was inconsistent with the previous studies by Pearce *et al*.^6^. The reason for this inconsistency may be attributed to differences in the study methods, sample collection, and geographical differences in patient. Based on the present result of predicting spontaneous PD during the second trimester of pregnancy in women in China, in our opinion, special attention should be given when a patient has two or more positive variables as defined in this study. In addition, some targeted interventions, such as the application of glucocorticoids for promoting fetal lung maturation or the administration of magnesium sulfate to protect the fetal central nervous system, should be performed if appropriate in the clinical situation. Of course, patients with only one positive variable for predicting PD cannot be ignored, as there was still a moderate accuracy rate of 42.9% with only one variable, so observation strict and regular evaluation of the development of the uterus and fetus is necessary to prevent the occurrence of PD.

In summary, we propose a model of predicting PD by introducing eight predictive indices originating from previous studies. The model showed an effective improvement in the sensitivity, specificity and predictive accuracy compared to previous models for predicting PD. In conclusion, the evaluation model of equal to or more than two positive variables provides a feasible reference for predicting PD in the second trimester of pregnancy in clinical patients. However, there are two limitations in this study. One limitation was the insufficient sample size for validation of the model because of other limitations of time, space and funding in the present study. The lack of sample size is particularly represented in the group with ≥four positive variables. The other limitation was that genetic factors were not included in the present model because of a lack of reference literature. In a future study, we will expand the number of samples by including more pregnant patients from more hospitals, and we will proceed to evaluate the role of genetic factors in the occurrence of PD, seeking to further improve the sensitivity, specificity and accuracy of the model and reduce false-positive results.

## Materials and Methods

### Establishment of the model

The study included two steps: establishment and verification of the model. When constructing the model, the test variables were selected discreetly according to the criteria of optimal sensitivity and specificity, which were determined based on previous multicenter or single-center studies of predicting PD in pregnant women from 1997 to 2016. Data involving sample size, cut-off values, sensitivity and specificity, from these studies was acquired using PubMed. As a result, ten variables from 17 original studies were included in the present model. The included variables were as follows: “history of PD, prepregnancy BMI, use of tocolytic agents, cervix tissue elasticity, densitometry of cervix, cervical dilatation, cervical length, fetal fibronectin in CVF, serum MIF, IL-1β in CVF”. Cervical phosphorylated insulin-like growth factor binding protein-1 (phIGFBP-1) testing^34^, plasma corticotrophin releasing hormone (CRH) levels^35^, placenta previa^10^ and bacterial vaginosis^36^ were excluded from the model because of the lower sensitivity or specificity than the included variables.

### Design of the protocol

For the verification of the model, the designed prospective study of enrolling pregnant patients was approved from the Medical Ethics Committees in Xiangya Hospital, The Second Xiangya Hospital and Weihai municipal hospital. All experiments were carried out in accordance with the Declaration of Helsinki. A total of 186 pregnant patients who made scheduled visits to three participating hospitals from January 1, 2015 to March 1, 2016 were enrolled in accordance with approved guidelines. All participants were informed and signed a consent form presented by trained interviewers and their general epidemiologic and clinical data were recorded. Their gestational age varied from 17 to 28 weeks and 6 days as determined by the last menstrual period and ultrasonography in the first or early second trimester. Cervicovaginal fluid or serum specimens were collected and measurements from cervical ultrasonography were performed during a routine speculum examination by the managing gynecologist. Additionally, the following conditions were considered for exclusion at the time of enrollment: age <15 years, multiple gestations (≥triple pregnancy), uterine or vaginal deformity, amniotic sac rupture, cervical dilatation ≥3 cm, serious infection of the genital tract, frequent symptoms of threatened abortion such as vaginal bleeding or uterine contractions, and obstetric complications such as hypertension and diabetes mellitus. PD was defined as the spontaneous or non-indicated preterm delivery or preterm premature rupture of the fetal membranes prior to 37 weeks of gestation, and the correspondent data were collected during a follow-up period.

### Measurement of ultrasound

Ultrasound scans of the uterine cervix were performed with a Premium Ultrasound System (5–9 MHz, Hi Vision Preirus, Hitachi Medical Systems, Wiesbaden, Germany). Measurements including cervical length via transvaginal ultrasonography, evaluation of the cervical tissue stiffness by sonoelastography and mean gray analysis were performed using quantitative ultrasound. The methods of detection and evaluation were determined by previous protocols^22, 24, 28^. Cervical length was defined as the distance between the internal and external os, which was measured in a sagittal plane of the cervix. Cervical tissue stiffness was represented by a color scale. Blue is indicative of stiff tissue, green represents average stiffness and red represents soft tissue. Furthermore, the proportion of blue area was calculated in a rectangular region of interest representing the posterior wall of the cervix. Acoustic densitometry (amplitude scale) of a region of constant size (10 Diameter Circle) in the cervical tissue was also measured using the US System (Philips Medical Systems, Hamburg, Germany). Each examination was repeated three times by two different investigators.

### Detection of cytokines

A total of 10 mL of cervicovaginal lavage fluid was collected, and the centrifugal supernatants were frozen at −70 °C for detecting the concentrations of IL-1β or fetal fibronectin (fFN) as previously described^14, 21, 33^. An IL-1β human enzyme-linked immunosorbent assay (ELISA) Kit (Thermo Fisher Scientific Inc, California, USA) and fFN Enzyme Immunoassay Kit (Adeza Biomedical Corporation, Sunnyvale, Calif) were used, and a positive result of IL-1β or fFN was defined as >55 pg/mL or ≥50 ng/mL, respectively. In addition, serum MIF was detected using a sandwich ELISA assay as described by Pearce *et al*.^30, 31^, and an anti-MIF polyclonal antibody (Abcam, Cambridge, USA) was used with a positive result defined as >9.2 ng/mL. All cytokines were determined repeatedly three times by each separate sample according to the manufacturer’s instructions.

### Evaluation of prediction efficiency

Positive or negative results of the test variables were calculated according to their corresponding cut-off values in each pregnant patient, and the difference in the distribution of positive variables between the PD group and the full-term delivery group was analyzed using the χ^2^ test with *P* < 0.05. Furthermore, sensitivity, specificity and accuracy rates of single or different combinations of positive variables were also calculated for evaluating the efficiency of predicting PD. Then, the prediction efficiency of more than one or several positive results including 2×, 3× or 4× were also evaluated. Ultimately, prediction efficiency and cut-off point of the established model was evaluated by a comprehensive comparison of sensitivity, specificity and accuracy rates among different combinations of positive variables.

### Data availability

All data generated or analyzed during this study are present in the article; Additional data related to this paper may be requested from the authors.
